# Cellular Mechanisms of Rejection of Optic and Sciatic Nerve Transplants: An Observational Study

**DOI:** 10.1097/TXD.0000000000001012

**Published:** 2020-07-24

**Authors:** Merve Yonar, Mayuko Uehara, Naima Banouni, Vivek Kasinath, Xiaofei Li, Liwei Jiang, Jing Zhao, Fengfeng Bei, Su Ryon Shin, Curtis L. Cetrulo, Nasim Annabi, Reza Abdi

**Affiliations:** 1 Transplantation Research Center, Renal Division, Brigham and Women’s Hospital/Harvard Medical School, Boston, MA.; 2 Department of Neurosurgery, Brigham and Women’s Hospital/Harvard Medical School, Cambridge, MA.; 3 Division of Engineering in Medicine, Brigham and Women’s Hospital/Harvard Medical School, Boston, MA.; 4 Department of Surgery, Massachusetts General Hospital/Harvard Medical School, Boston, MA.; 5 Chemical and Biomolecular Engineering Department and Center for Minimally Invasive Therapeutics (C-MIT), California NanoSystems Institute (CNSI), University of California—Los Angeles, Los Angeles, CA.

## Abstract

**Background.:**

Organ transplantation is a standard therapeutic strategy for irreversible organ damage, but the utility of nerve transplantation remains generally unexplored, despite its potential benefit to a large patient population. Here, we aimed to establish a feasible preclinical mouse model for understanding the cellular mechanisms behind the rejection of peripheral and optic nerves.

**Methods.:**

We performed syngenic and allogenic transplantation of optic and sciatic nerves in mice by inserting the nerve grafts inside the kidney capsule, and we assessed the allografts for signs of rejection through 14 d following transplantation. Then, we assessed the efficacy of CTLA4 Ig, Rapamycin, and anti-CD3 antibody in suppressing immune cell infiltration of the nerve allografts.

**Results.:**

By 3 d posttransplantation, both sciatic and optic nerves transplanted from BALB/c mice into C57BL/6J recipients contained immune cell infiltrates, which included more CD11b^+^ macrophages than CD3^+^ T cells or B220^+^ B cells. Ex vivo immunogenicity assays demonstrated that sciatic nerves demonstrated higher alloreactivity in comparison with optic nerves. Interestingly, optic nerves contained higher populations of anti-inflammatory PD-L1^+^ cells than sciatic nerves. Treatment with anti-CD3 antibody reduced immune cell infiltrates in the optic nerve allograft, but exerted no significant effect in the sciatic nerve allograft.

**Conclusions.:**

These findings establish the feasibility of a preclinical allogenic nerve transplantation model and provide the basis for future testing of directed, high-intensity immunosuppression in these mice.

Organ transplantation has emerged as a lifesaving treatment for patients with irreversible organ damage. Kidney, heart, face, and limb transplantations have entered into clinical practice, but the utility of nerve transplantation has thus far been limited.^1-4^ Notably, the size of the patient population who could benefit from peripheral or optic nerve transplantation is extremely high. Currently, 285 million people worldwide suffer from visual impairment, of whom 14% (39 million) are blind.^5^ Diabetic retinopathy, age-related macular degeneration, and glaucoma are the major causes of irreversible blindness,^6-8^ although trauma and optic nerve tumors represent important origins as well.^9,10^ Traumatic eye injury and other visual problems are the fourth most common wounds occurring in the battlefield, and currently 158 000 living veterans have blindness in the United States.^11^ The pathogenesis behind irreversible blindness is marked by the inability of retinal ganglion cells to regenerate.^5,12^ Recent studies have focused on the prospect of whole eye transplantation, but the main challenges to the success of this operation are rejection and survival of the optic nerve.^12^

Moreover, every year, approximately 13–23 out of 100 000 people are confronted with peripheral nerve injuries.^11,13^ Trauma, tumors, and iatrogenic lesions are the leading causes of peripheral nerve injury,^14^ and the main treatment options for small injuries are either primary suture, creation of a nerve conduit, or replacement with an autologous nerve graft, depending on the severity of the injury.^15-17^ However, major injuries that cause longer disruptions of the nerve have limited therapeutic options.^18^ Allogeneic nerve transplantation could be an ideal option to bridge long gaps in the injured nerve, but the paucity of basic information of the cellular mechanisms of rejection and standard preclinical studies identifying immunosuppressive regimens have hampered the development of nerve transplantation.^19,20^ Some past attempts to understand the immunologic basis for rejection of allogeneic nerve transplants have been undertaken, but the comprehensive characterization of these cellular mechanisms remains understudied.^21-23^ In addition, once a feasible model of allogeneic nerve transplantation has been established, a detailed understanding of the cellular mechanisms will assist in the identification of a suitable immunosuppressive therapy. Herein, we sought first to establish a feasible preclinical model of allogeneic nerve transplantation for the examination of peripheral and optic nerve rejection as well as characterization of the immunologic response against the nerve allografts; then, we endeavored to examine the effect of immunosuppression on features of their rejection.

## MATERIALS AND METHODS

### Animals

C57BL/6J (H-2b) and BALB/c (H-2d) mice were purchased from Jackson Laboratories (Bar Harbor, ME). Adeno-associated virus-2 was a generous gift from Dr Fengfeng Bei. All animal experiments and methods were carried out in accordance with approved guidelines and were approved by the Institutional Animal Care and Use Committee of Brigham and Women’s Hospital, Harvard Medical School.

### Optic and Sciatic Nerve Transplantations

Optic and sciatic nerves were procured and kept in University of Washington solution at 4°C until implantation. A self-retractor was placed in the abdomen following the abdominal incision and the kidneys were exposed. The kidney capsule was held with sharp tweezers while a ~2-mm incision was made. The optic or sciatic nerve was inserted gently under the kidney capsule through the opening.

### Heterotopic Cardiac Transplantation

Heterotopic intra-abdominal cardiac transplantation was performed using microsurgical techniques, as described previously.^24^ Donor’s hearts were procured and kept in University of Washington solution at 4°C. Then, they were transplanted intra-abdominally within 30 min after procurement. The survival of cardiac grafts was assessed by daily palpation.

### Immunosuppression

CTLA4 Ig, Rapamycin (Cayman Chemical Company), and anti-CD3 antibody (rabbit monoclonal anti-CD3 [SP7]) (Abcam) were used for immunosuppression. Five hundred micrograms of CTLA4 Ig were injected intraperitoneally into recipient mice on the day of transplantation (day 0), and 250 μg were injected on days 2, 4, 6, and 8. Seventy-five micrograms of Rapamycin were injected intraperitoneally into recipient mice on days 0, 1, 2, and 3. Fifty micrograms of anti-CD3 were injected into mice intravenously daily, starting a day before transplantation until day 3, following a protocol used previously for heart transplantation.^25^

### Immunofluorescent Staining and Hematoxylin and Eosin

Kidneys containing the transplanted nerves were procured from mice at the time of euthanizing. The kidney samples were either fixed in 10% formalin and embedded in paraffin blocks for hematoxylin and eosin staining (H&E) or cryoprotected in OCT compound (Tissue-Tek, Thermo Fisher Scientific) for immunofluorescence staining. A cryostat was used to produce 6-μm-thick flash frozen tissue sections (Leica Cm 1510 S). The sections were fixed in ice-cold acetone for 5 min and incubated with a blocking solution containing 3% BSA for 30 min at room temperature. Then, the sections were incubated with primary antibodies [anti-CD3 (Abcam), anti-B220 (BD Bioscience), anti-CD11b (BioLegend)] for 1 h at room temperature or overnight at 4°C. After washing with DPBS (Dulbecco’s PBS), the sections were incubated for 30 min with AlexaFluor 594 goat anti-rabbit IgG, AlexaFluor 488 goat anti-rat IgG, AlexaFluor 594 goat anti-rat IgG, and AlexaFluor 594 donkey anti-goat IgG secondary antibodies, respectively, to bind to the primary antibodies. DAPI (Vectashield) was used to stain nuclei.

### Mean Fluorescence Intensity Measurement of CD3^+^, CD11b^+^, B220^+^ Signals

We used Image J software to measure the mean fluorescence intensity (MFI) of the CD3^+^, CD11b^+^, and B220^+^ signals in the fluorescence micrographs of nerve allografts. First, we measured the areas of CD3^+^, CD11b^+^, and B220^+^ signals accordingly and divided these by the area of DAPI signal to calculate the percentage of each signal.

### Flow Cytometry

Flow cytometry was used to evaluate the composition of nerves and to quantify immune cells in the secondary lymphoid organs. Nerves were dissected into small pieces and then digested at 37°C by an enzyme mix composed of 0.1 mg/mL DNAse (Roche), 0.2 mg/mL collagenase P (Roche), and 0.8 mg/mL dispase II (Sigma-Aldrich). Following digestion, the cell suspension was centrifuged at 1600 rpm for 5 min, the supernatant was discarded, and the pellet was resuspended in media (DMEM-Lonza Bioscience). Then, the single cell suspensions were placed into 96-well V-bottom plates (Corning Incorporated, Corning, NY) for surface staining. First, the cells were stained with eBioscience Fixable Viability Dye eFluor780 diluted 1:1000 in DPBS for 30 min at 4°C. Next, the cells were washed with fluorescence-activated cell sorting (FACS) buffer (DPBS + 2% fetal bovine serum + 1 mmol/L EDTA + 0.1% sodium azide) and incubated with major histocompatibility complex (MHC) class I, MHC class II, PD-L1, CD90, CD105, CD44, CD29, and CD73 antibodies (BioLegend) for 25 min at 4°C. Then, the cells were washed with FACS buffer again and fixed in a solution containing FACS buffer + 1% formalin. Finally, the cells were processed by the flow cytometer (BD FACSCanto II).

### Histological Assessment

To compare the effect of immune therapeutics on optic and sciatic nerve allografts, we evaluated immune cell infiltration histologically as below. Five-μm-thick, formalin-fixed, paraffin-embedded kidney sections containing sciatic nerve or optic nerve were stained with H&E. Lymphocyte infiltration was scored blindly from 0 to 3 in 4 random fields of each H&E section (3 sections per nerve, 7 mice for control, and 3 mice per treatment group). The scores were defined as follows: 0, no cellular infiltration; 1, mild cellular infiltration; 2, moderate cellular infiltration; and 3, severe cellular infiltration.

### Mixed Lymphocyte Reaction

An allogeneic 1-way mixed lymphocyte reaction (MLR) technique was used to compare the immunogenicity of sciatic and optic nerves. In order to create an allogenic environment, we used tissues from BALB/c mice as stimulators and C57BL/6 splenocytes from either sciatic nerve transplant or optic nerve transplant mice as responders. Sciatic nerves, optic nerves from BALB/c mice, and spleens from C57BL/6 mice were digested as described above and suspended in complete DMEM 1× medium (Corning; Manassas, VA) supplemented with 10% fetal bovine serum (Gemini Bio-Products), 1% penicillin/streptomycin (Corning), and 1% L-Glutamine (Corning). Ten sciatic nerves and 10 optic nerves from 5 mice were pooled separately. Cells of BALB/c origin were irradiated (3 min, 10–15 × 10^3^ cGy of radiation). Then, an equal number (500 000 cells/well) of sciatic nerve cells and optic nerve cells were added into a 96-well round-bottom cell culture plate (Corning Incorporated, Corning, NY) and co-cultured with an equal number of splenocytes from C57BL/6 mice. After a 48-h incubation period, the cells were labeled with 0.5 μCi [^3^H]-Thymidine per well (PerkinElmer). After 16 h of incubation, the cell suspensions were aspirated on a Filtermat A glass fiber filter mat (PerkinElmer) with a 96-well microplate Harvester96 Mach III cell harvester by Tomtec, and sealed in a plastic sample bag (PerkinElmer) soaked with Betaplate Scint liquid scintillation cocktail (PerkinElmer). Then, [^3^H]-Thymidine incorporation counts per minute were acquired with a WALLAC Microbeta TriLux Liquid Scintillation and Luminescence Counter (PerkinElmer).

### Luminex Assay

The levels of cytokines and chemokines were measured in the media retrieved from the MLR assay, using the Milliplex magnetic kit (EMD Millipore Corporation) as per the manufacturer’s instructions.

### Statistical Analysis

Data are represented as mean ± SEM. Statistical analysis was performed using the unpaired, 2-tailed Student’s *t*-test or 1-way ANOVA test. *P* values of <0.05 were considered statistically significant (*P < 0.05, **P < 0.01, ***P < 0.001).

## RESULTS

### Autologous Optic and Sciatic Nerve Transplantation

**FIGURE 1.**
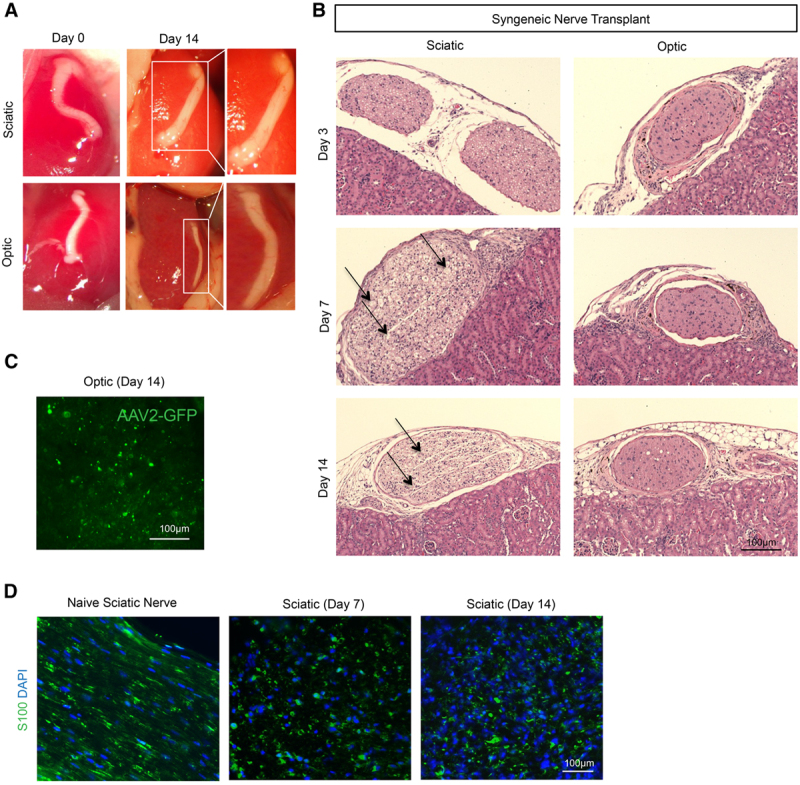
Autologous optic and sciatic nerve transplantation. A, Representative photographs demonstrate a macroscopic appearance of nerve grafts on d 0 and 14 following autologous transplantation of sciatic and optic nerves between genetically identical C57BL/6J mice. B, Light micrographs of H&E-stained sciatic and optic nerve autografts on d 3, 7, and 14. Arrows indicate allograft regions affected by vacuolization. Images are representative of 5 independent experiments (n = 5). C, Following intraophthalmic injection of GFP-labeled adeno-associated virus-2 before procurement of optic nerve, fluorescence micrograph of optic nerve autograft on d 14 demonstrates the presence of GFP, confirming the presence of axons. D, Fluorescence micrograph of S100^+^ Schwann cells (green) in naïve sciatic nerve and sciatic autografts on d 7 and 14 demonstrate persistence of S100^+^ signal. AAV2-GFP, GFP-labeled adeno-associated virus 2; H&E, hematoxylin and eosin.

Autologous optic and sciatic nerve transplantation was performed using C57BL/6J mice. The autografts were placed under the recipient’s kidney capsule, a suitable place because of its highly vascular structure. Fourteen days after transplantation, both optic and sciatic nerve autografts were observed visually to be intact with a slight increase in surrounding vascularization, but without any change in the size or shape of the nerve autografts (Figure 1A). The histological appearance of both autografts on day 3 revealed an absence of infiltration or structural changes. However, vacuolization in the sciatic autograft was noted at day 7 and progressed over time through day 14, though no infiltration was identified (Figure 1B). Interestingly, the anatomical structure of the optic nerve autografts was preserved better than the sciatic autografts, as evidenced by less severe vacuolization at all time points (Figure 1B). To confirm the presence of axons in the optic nerve autograft, we injected GFP-labeled adeno-associated virus 2 (AAV2-GFP), which is known to bind and transfect retinal cells without any significant pathogenicity into the donor, intraophthalmically, before retrieving the nerves.^26^ By day 14, a positive GFP signal was identified in the optic nerve autograft (Figure 1C). We also found that Schwann cells were well-preserved and abundant in the sciatic nerve autograft, even at day 14 (Figure 1D).

### Allogeneic Optic and Sciatic Nerve Transplantation

**FIGURE 2.**
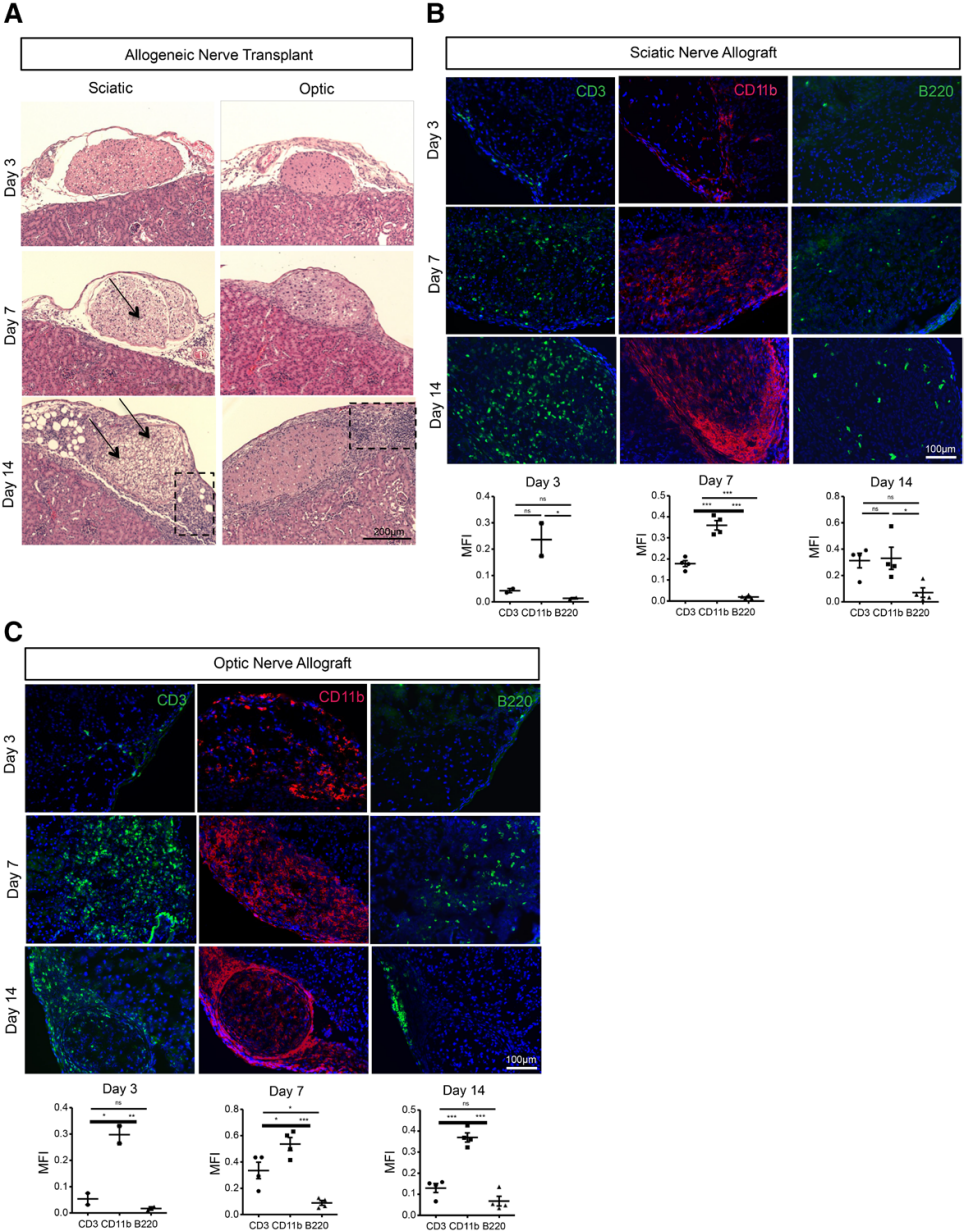
Allogeneic optic and sciatic nerve transplantation and characterization of immune cells. A, H&E images of sciatic and optic grafts on d 3, 7, and 14 after allogeneic transplantation between BALB/c and C57BL/6J mouse. Arrows indicate allograft regions affected by vacuolization, and boxes indicate areas of immune cell infiltration. Images are representative of 5 independent experiment (n = 5). B, Representative fluorescence micrographs of CD3^+^ T cells (green), B220^+^ B cells (green), and CD11b^+^ cells (red) in sciatic nerve allografts at d 3, 7, and 14 following transplantation from BALB/c mouse into C57BL6/J recipient (n = 2 at d 3, n = 4 at d 7 and 14). Comparisons of MFI of CD3^+^, B220^+^, and CD11b^+^ signals in the sciatic nerve allograft at d 3 (n = 2), d 7 (n = 4), and d 14 (n = 4). C, Representative fluorescence micrographs of CD3^+^ T cells (green), B220^+^ B cells (green), and CD11b^+^ macrophages (red) in optic nerve allografts at d 3, 7, and 14 following transplantation from BALB/c mouse into C57BL6/J recipient (n = 2 at d 3, n = 4 at d 7 and 14). Comparisons of MFI of CD3^+^, B220^+^, and CD11b^+^ signals in the optic nerve allograft at d 3 (n = 2), d 7 (n = 4), and d 14 (n = 4). Data are shown as mean ± SEM; student *t*-test. H&E, hematoxylin and eosin; MFI, mean fluorescence intensity.

After establishing the viability of subcapsular placement of sciatic and optic nerve autografts, we repeated the same experiment, substituting allogeneic nerves for the autografts. Sciatic and optic nerves were retrieved from BALB/c mice and implanted under the kidney capsule of C57BL/6J mice. At day 3, the structure of both the sciatic and optic nerves appeared intact. At day 14, the sheaths around both the optic and sciatic allografts remained continuous and intact, but a large amount of immune cell infiltrates were noted in the vicinity of the nerves. Similar to the phenomenon observed in the autografts, vacuolization was more abundant in the sciatic allografts in comparison with optic allografts (Figure 2A).

Next, we sought to identify the immune cells that play a major role in nerve rejection. At day 3, scant infiltrates of CD3^+^ T and B220^+^ B cells were identified in the sciatic allograft, whereas larger aggregates of CD11b^+^ macrophages were observed. The density of infiltrating immune cells increased over time at day 14 (Figure 2B). We assessed the size of the cellular infiltrates at days 3, 7, and 14 through calculation of the MFI of the CD3^+^, CD11b^+^, and B220^+^ signals, which demonstrated at all time points that CD11b^+^ cells were the most abundant, and B220^+^ B cells were the least abundant (Figure 2B). Similar to sciatic nerve allografts, optic nerve allografts also showed more abundant CD11b^+^ cells than CD3^+^ T cells or B220^+^ B cells (Figure 2C), as evidenced by assessment of these 3 immune cells infiltrates by MFI (Figure 2C).

### Assessment of Alloimmune Response in Secondary Lymphoid Organs of Nerve Allograft Recipient Mice

**FIGURE 3.**
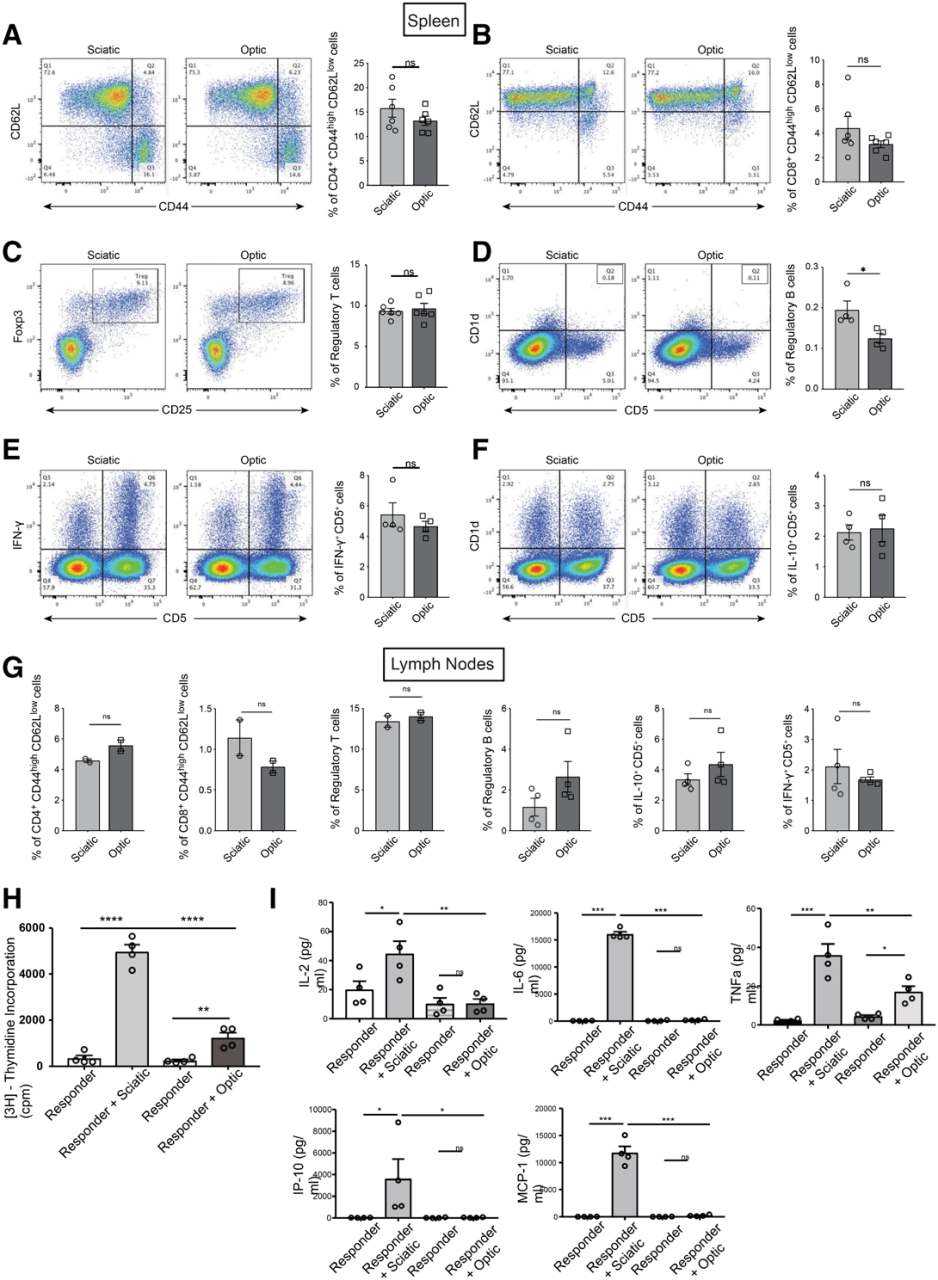
Assessment of alloimmune response in secondary lymphoid organs of nerve allograft recipient mice. A–F, Comparisons of the (A) CD44^high^ CD62L^low^ CD4^+^ effector memory T cell (15.77% vs 13.22%; *P* = 0.2454), (B) CD44^high^ CD62L^low^ CD8^+^ effector memory T cell (4.405% vs 3.092%; *P* = 0.2155), (C) CD25^+^ FOXP3^+^ regulatory T cell (9.655% vs 9.332%; *P* = 0.6666), (D) B220^+^ CD1d^+^ CD5^+^ regulatory B cell (0.1950% vs 0.1250%; *P* = 0.0304), (E) IFN-γ^+^ CD5^+^ cell (5.423% vs 4.658%; *P* = 0.3924), and (F) IL-10^+^ CD5^+^ cell (2.128% vs 2.250%; *P* = 0.8137) populations between the spleens of mice that have received either optic or sciatic nerve allografts. G, Comparisons of the CD44^high^ CD62L^low^ CD4^+^ effector memory T cell (4.580% vs 5.570%; *P* = 0.1209), CD44^high^ CD62L^low^ CD8^+^ effector memory T cell (1.140% vs 0.7850%; *P* = 0.2662), CD25^+^ FOXP3^+^ regulatory T cell (13.40% vs 14.00%; *P* = 0.5577), B220^+^ CD1d^+^ CD5^+^ regulatory B cell (1.160% vs 2.648%; *P* = 0.1392), IFN-γ^+^ CD5^+^ cell (2.113% vs 1.685%; *P* = 0.4818), and IL-10^+^ CD5^+^ cell (3.355% vs 4.350%; *P* = 0.2963) populations between the lymph nodes of mice that have received either optic or sciatic nerve allografts. Data were collected from 4 independent experiments and shown as mean ± SEM; student *t*-test. H, Mixed lymphocyte reaction (MLR) assay with splenocytes from transplanted mice (sciatic or optic) and nerve cells (sciatic and optic) from donor mice (BALB/c). The splenocytes from the sciatic nerve-transplanted group showed significantly higher proliferation compared with the optic nerve-transplanted group (responder+sciatic vs responder+optic, 4967 ± 312.5 vs 1237 ± 222.5, ***P < 0.001, n = 4/group). I, Luminex assay showed significantly higher inflammatory cytokines and chemokines in the media taken from the sciatic nerve-transplanted group in comparison with the optic nerve-transplanted group (responder+sciatic vs responder+optic, 44.6 ± 8.6 vs 10.4 ± 3.1, **P < 0.01 for IL-2, 16 065 ± 463.1 vs 213.1 ± 56.9, ***P < 0.001 for IL-6, 35.8 ± 5.8 vs 16.9 ± 2.9, **P < 0.01 for TNFα, 3605 ± 1828 vs 45.5 ± 13.8, *P < 0.05 for IP-10, 11 793 ± 1196 vs 218.9 ± 64.2, ***P < 0.001 for MCP-1, n = 4/group). MCP-1, monocyte chemoattractant protein-1.

In order to compare the activation of the systemic immune response between the recipients of optic and sciatic nerve allografts, we analyzed the immune cells from the spleens and kidney-draining lymph nodes by flow cytometry. No significant difference was identified in the percentages of CD4^+^ effector memory T cells, CD8^+^ effector memory T cells, CD4^+^FoxP3^+^CD25^+^ regulatory T cells, IFN-γ^+^ CD5^+^ cells, and IL-10^+^ CD5^+^ cells in the spleen (Figure 3A–F). Interestingly, B220^+^ CD1d^+^ CD5^+^ regulatory B cells were significantly more abundant in the spleen of optic allograft recipient mice (Figure 3D), but no difference was noted in the kidney-draining lymph nodes (Figure 3G). No significant difference in the percentages of effector memory T cells, regulatory T cells, IFN-γ^+^ CD5^+^ cells, and IL-10^+^ CD5^+^ cells in the lymph nodes was observed as well (Figure 3G).

Furthermore, we assessed the alloreactivity of the splenocytes from transplanted mice using an MLR assay. Sciatic or optic nerves from BALB/c mice were transplanted into C57BL/6 mice. Then, their splenocytes (responder) were cultured at 7 d posttransplant with either irradiated sciatic or optic nerve cells (stimulator) retrieved from BALB/c mice. The proliferation rate in the splenocytes from the sciatic nerve responder group was higher in comparison with the optic nerve responder group (Figure 3H). We also used the media from these samples to perform a Luminex assay, and we found that the levels of proinflammatory cytokines, such as IL-2, IL-6 and TNFα, and chemokines, including IP-10 and monocyte chemoattractant protein-1 (MCP-1), were significantly higher in the sciatic nerve responder group than the optic nerve responder group (Figure 3I).

**FIGURE 4.**
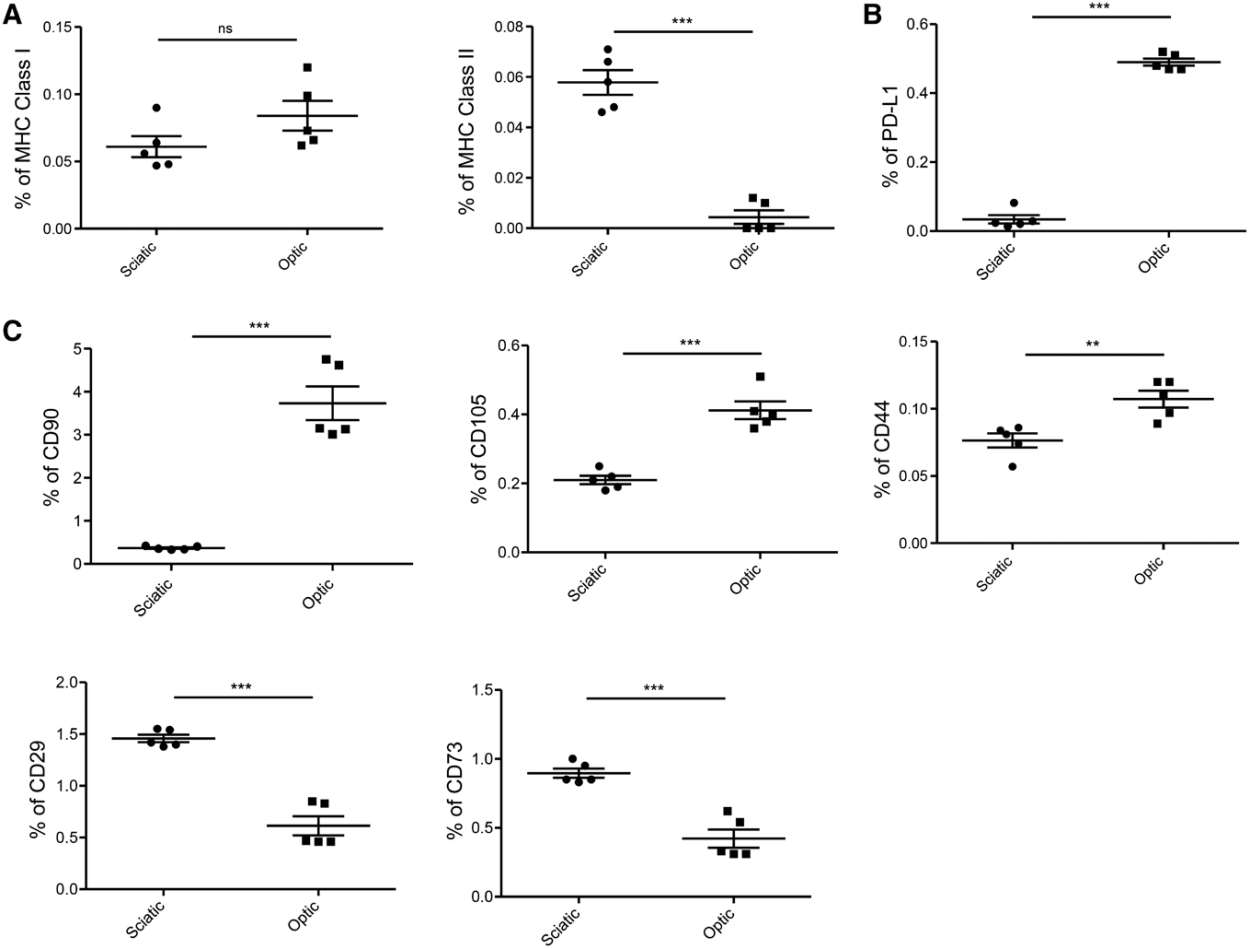
Assessment of sciatic and optic nerve by flow cytometry. A, Comparison of percentages of MHC Class I and MHC Class II (sciatic vs optic, 0.06% vs 0.08%, *P* = ns for MHC Class I, 0.06% vs 0.004%, ***P < 0.001 for MHC Class II, n = 5/group). B, Optic nerve showed significantly higher PD-L1 population (sciatic vs optic, 0.03% vs 0.4%, ***P < 0.001, n = 5/group). C, Optic nerve showed significantly higher population of CD90, CD105, and CD44 compared with sciatic nerve (sciatic vs optic, 0.3% vs 3.7%, ***P < 0.001 for CD90, 0.2% vs 0.4%, ***P < 0.001 for CD105, 0.07% vs 0.11%, **P < 0.01 for CD44, n = 5/group). CD73 and CD29 were less in optic nerve compared with sciatic nerve (sciatic vs optic, 0.4% vs 0.2%, ***P < 0.001 for CD73, 0.52% vs 0.24%, ***P < 0.001 for CD29, n = 5/group). MHC, major histocompatibility complex.

To gain greater insight into the principles guiding the immunogenicity of the 2 nerve types, we analyzed the population of cells expressing MHC class-I and class-II, as well as several stromal cell markers in both nerves. Flow cytometric analysis revealed no significant difference in the expression of MHC class-I between the optic and the sciatic nerves, but the expression of MHC class-II was significantly lower in the optic nerve than the sciatic nerve (Figure 4A). Then, we examined the expression of the key negative costimulatory molecule PD-L1, which was markedly higher in the optic nerve than the sciatic nerve (Figure 4B). The immunomodulatory effects of stromal cells have been well established.^27,28^ Therefore, we examined stromal cell markers, and we found a significantly higher population of CD90^+^, CD105^+^, and CD44^+^ cells, but a lower population of CD29^+^ and CD73^+^ cells in the optic nerve in comparison with the sciatic nerve (Figure 4C).

### Identifying an Effective Immunosuppressive Regimen for Reduction of Intragraft Inflammation

**FIGURE 5.**
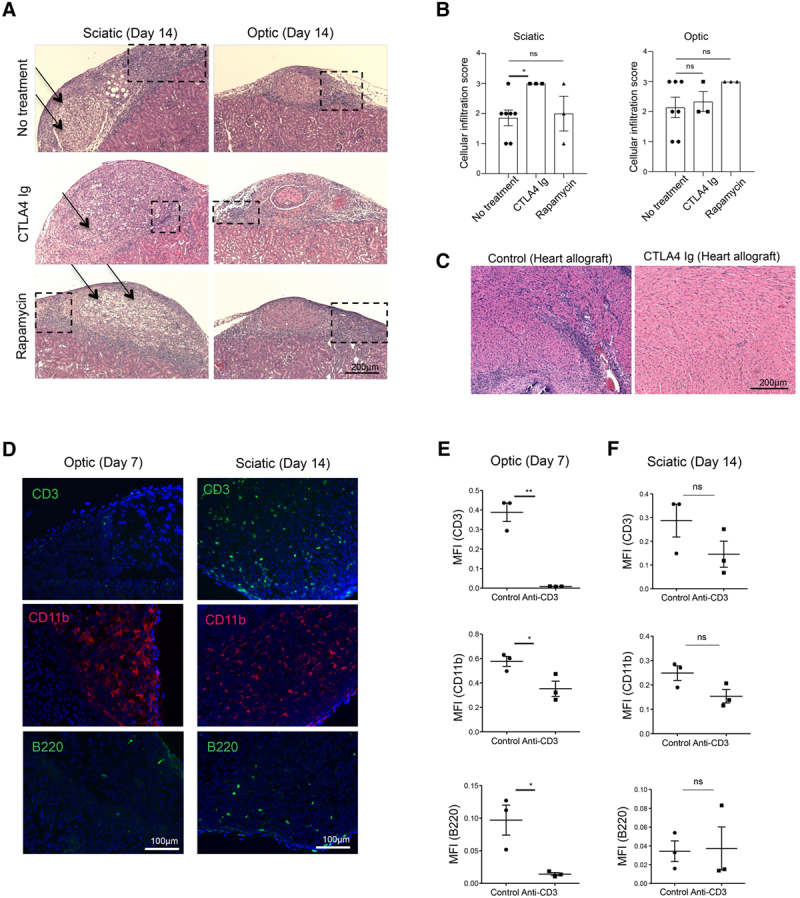
Identification of effective immunosuppressive regimens for prevention of nerve allograft rejection. A, Representative light micrographs of H&E-stained nerve allografts demonstrate the comparison between the histologic appearance at d 14 of the untreated control group and nerve allografts from mice treated with CTLA4 Ig or Rapamycin. Arrows indicate allograft regions affected by vacuolization, and boxes indicate areas of immune cell infiltration. B, Comparisons of cellular infiltration scores at d 14 between sciatic nerve allografts or optic nerve allografts from untreated mice and those from mice treated with CTLA4 Ig or Rapamycin. C, Representative light micrographs of H&E-stained heart allografts demonstrate a comparison between the histologic appearance of the untreated control group and allografts from mice treated with CTLA4 Ig. D, Representative fluorescence micrographs of CD3^+^ T cell (green), B220^+^ B cell (green), and CD11b^+^ macrophage (red) populations in optic nerve allograft at d 7 following treatment with anti-CD3 and sciatic nerve allograft at d 14 following treatment with anti-CD3. E, Comparison of MFI of CD3^+^ (0.3883% vs 0.0084%, **P < 0.01; n = 3), B220^+^ (0.0970% vs 0.0142%, *P < 0.05; n = 3), and CD11b^+^ signals (0.5763% vs 0.3527%, *P < 0.05; n = 3) between sciatic nerve allografts from untreated mice and those from mice treated with anti-CD3. F, Comparison of MFI of CD3^+^ (0.2874% vs 0.1457%, *P* = ns; n = 3), B220^+^ (0.0342% vs 0.0372%, *P* = ns; n = 3), and CD11b^+^ signals (0.2488% vs 0.1537%, *P* = ns; n = 3) between optic nerve allografts from untreated mice and those from mice treated with anti-CD3. Data are shown as mean ± SEM, student *t*-test. H&E, hematoxylin and eosin; MFI, mean fluorescence intensity.

Finally, we treated nerve allograft recipient mice with different immunosuppressive drugs to identify optimal immune therapeutic strategies that can be used in the future to halt nerve rejection. Following transplantation, multiple dosages either of CTLA4 Ig or Rapamycin were given to recipient mice. Both treatment regimens failed to protect allografts of both nerves from immune cell infiltration (Figure 5A). Interestingly, sciatic allografts from the mice treated with CTLA4 Ig contained more severe immune infiltration in comparison with the control groups (Figure 5B). In contrast, the immune cell infiltrates in the optic allografts from the mice treated with either CTLA4 Ig or Rapamycin were not significantly different from the control groups (Figure 5B). The same treatment dose of CTLA4 Ig resulted in tolerance of heart allografts (compared with control without treatment retrieved at day 7) (Figure 5C). However, treatment with anti-CD3 reduced markedly the CD3^+^ T cell, B220^+^ B cell, and CD11b^+^ macrophage populations in the optic nerve allograft (Figure 5D and E). Interestingly, anti-CD3 did not exhibit the same protective effect for the sciatic nerve allograft (Figure 5D and F).

## DISCUSSION

Because of the first successful kidney transplant, a significant effort has been made to expand the utility of transplantation to other organs, including liver, pancreas, and vascularized composite tissue transplants.^29-31^ However, the field of nerve transplantation remains relatively unexplored, despite the remarkable potential therapeutic utility of peripheral and optic nerve allografts for a large patient population for whom nerve transplantation can have a major impact. Autologous peripheral nerve transplantation has been employed to bridge small gaps in peripheral nerve tracks, but autologous nerve tissue cannot be used for a complete repair of longer nerve injuries, because of lack of adequate tissue.^15^ Therefore, allogeneic nerve transplantation is 1 possible solution to this problem. However, the effect of alloimmune reactions against nerve tissue is still unknown.

Eye transplantation remains a monumental challenge, for which the rejection of optic nerve remains a key barrier to success. Indeed, prior articles written by Washington and colleagues have established that failure of regeneration and rejection of the optic nerve are major factors that must be overcome in order for eye transplantation to succeed.^12,32^

Prerequisite to the success of nerve transplantation is a better understanding of the cellular mechanisms that lead to nerve rejection and identification of optimal immunosuppressive regimens to reduce alloimmune responses. Our nerve transplant model provides us with the ability to perform these investigations, although it also has several limitations, such as the inability to test graft function due to technical restrictions.

In an attempt to unveil the cellular mechanisms behind the rejection of nerve transplants as well as to test the effect of common immunosuppressive agents on intragraft inflammation, we developed a mouse model of allogenic nerve transplantation, in which we transplanted optic and sciatic allografts under the kidney capsule. Models that rely on the implantation of allograft tissues under kidney capsule recapitulate alloimmunity from the perspective of allorecognition. A classic example is the transplantation of pancreatic islets or other implanted tissues, by which investigators have identified key pathways that promote rejection effectively and discovered tolerogenic drugs.^33-35^ These pathways overlap with those that are responsible for the rejection of vascularized organs under the kidney capsule. Our results demonstrate that implanting nerves under the kidney capsule represents a useful technical approach to maintain a viable organ, particularly with respect to the optic nerve. Although this site of transplantation may not provide the ability to test the full functionality of implanted nerves, it does provide useful information regarding the status of rejection and alloreactive immune cells infiltrating the nerves. In addition, vascularization of these grafts could impact various layers of tissues differently from implanting a large vascularized nerve. “Vacuolization” in axons occurs commonly following damage,^36,37^ and its extent correlates often with the severity of the damage. Mild damage may result in the formation of small and often reversible vacuoles in the axons. However, severe damage, such as allograft rejection in our model, may lead to widespread, large vacuoles, which were demonstrated in the allogeneic sciatic nerve allograft at day 14, and eventually to dissolution of the tissue. Thus, the presence of vacuolization could function as a histological “read-out” of the extent of nerve rejection.

We have demonstrated previously the importance of ischemia–reperfusion injury (IRI) to the development of chronic allograft rejection in human recipients of kidney allografts.^38^ We have also shown that allograft-resident dendritic cells (DCs) in the allografts upregulate their antigen presentation markedly under oxidative stress.^39-41^ Importantly, our group and others have revealed that ischemic DCs produce a large amount of IL-6, a key inflammatory cytokine that downregulates regulatory T cells (Treg), as it potentiates alloreactive CD4^+^ T cells.^41-43^ The extent to which IRI of the nerve allograft contributes to its immunogenicity and the vacuolization we observe in its axons is unclear and bears further study.

Histological quantification of the immune cell infiltrates identified by immunofluorescence indicated that both sciatic and optic nerve allografts contained CD11b^+^ cell infiltrates at 3 d posttransplantation, which increased over time to day 14. Future investigation is required to determine the proportion of these Cd11b+ cells that are proinflammatory M1 and anti-inflammatory M2 macrophages. We also observed a high amount of CD3^+^ T cell infiltrates, but very few B220^+^ cells were found in either nerve allograft.

We were not able to detect any difference in the peripheral immune responses by using standard techniques, such as flow cytometry for quantifying the activity of alloreactive T cells. This negative finding could have occurred either because of the sensitivity of these assays or the timing of retrieval of the lymphoid tissues. However, examination of the alloimmune responses in the periphery by MLR showed clearly higher proliferation of splenocytes from sciatic nerve-transplanted mice than optic nerve-transplanted mice. Luminex assay performed on their media revealed high levels of proinflammatory cytokines, such as IL-2, IL-6, and TNFα, in the sciatic nerve-transplanted mice. IP-10 (CXCL10) is produced by mature DCs,^44^ and MCP-1 is expressed by various cells in response to TNFα or other proinflammatory cytokines.^45,46^ The high levels of these cytokines and chemokines in sciatic nerve-transplanted mice indicate more severe alloimmunity in comparison with optic nerve-transplanted mice, which is also reflected by the histological observation of vacuolization in the allogeneic sciatic nerve graft. Because we observed a more severe alloimmune response in sciatic nerve-transplanted mice, we then examined the stromal composition of these nerves to understand better the etiology behind the higher immunogenicity of the sciatic nerve allograft. Interestingly, we found that the expression of MHC class II was significantly lower in the optic nerve, and the optic nerve expressed a higher amount of PD-L1, which is an important immunoregulatory marker. Furthermore, the optic nerve contained a significantly higher population of CD90^+^, CD105^+^, and CD44^+^ cells than sciatic nerve, which are markers of mesenchymal stem cells (MSCs), multipotent cells that harbor the potential to differentiate into a variety of cell types.^27^ Previous studies reported that MSCs perform a critical role in the suppression of immune responses.^27,47^ Recent studies have also focused on cancer stem cell markers, which mostly overlap with MSC markers, and these CD90-, CD105-, CD44-, CD27-, and CD73-expressing cells represent immunoevasion. In particular, CD90^+^ cell and CD44^+^ cell populations have received special attention for their potential role in immunoevasion.^48-51^ Therefore, the higher populations of CD90^+^ and CD44^+^ stromal cells we observed in the optic nerve suggest a higher immunoprivileged state in comparison with the sciatic nerve. This unique characteristic of the optic nerve may be supported by our MLR data, which showed that sciatic nerve cells were more immunogenic, and our Luminex data, which demonstrated lower cytokines and chemokines in the optic nerve-transplanted group. These data for the first time reveal the difference in the alloimmune responses seen toward these 2 types of nerves.

Once a suitable method for successful nerve transplantation has been established, the next challenge will be to identify the best immunosuppressive treatment to protect the nerve allograft from rejection. Strikingly, nerve allografts showed resistance to standard immunosuppressive regimens due to reasons that remain to be fully explored. Notably, the increase in the number of cell infiltrates observed following the CTLA4 Ig treatment of sciatic nerve allografts could be due to the potential deleterious effect of CTLA4 Ig in the reduction of regulatory T cell populations.^52-54^ Treatment with anti-CD3 cleared almost all of the T cells and B cells and decreased significantly the number of macrophages in the optic nerve allografts, possibly due to an overall reduction of nonspecific inflammatory responses and a decline of chemokines responsible for the trafficking of macrophages. The same treatment protocol demonstrated significant protection of allogeneic heart allografts.^25^

One of the limitations of our study was the lack of full characterization of macrophages and T cells (including tissue resident memory cells) for their regulatory and inflammatory features. Thus far, retrieving cells from these grafts has been extremely challenging technically. In our future studies, we are very much keen to retrieve immune cells from these grafts for full characterization using multicolor flow cytometry. These studies will also allow for proper quantification of inflammatory cells and robust comparisons of the severity of rejection between these 2 types of nerves. We are also planning to develop alternative plans to assess the function of these nerves that have been implanted inside the kidney capsule. Despite these limitations, it is noteworthy that that our method of intracapsular implantation will be extremely useful to address other facets of nerve transplantation, such as nerve regeneration and regrowth. In addition, this model can address the impact of IRIs on the nerves. We could potentially utilize this model as an ex vivo biorepository of nerve scaffold for use in the support of the transplantation of actual nerve. To our knowledge, no one has been able to demonstrate the lengthening of survival of an optic nerve transplant to the duration that we have shown. In summary, these data suggest that the optic nerve allograft is more immunoprivileged than the sciatic nerve allograft. Allogeneic nerve transplants may necessitate treatment with more potent immunosuppressive medications, such as T cell-depleting agents or combinatorial strategies, in which antimacrophage therapies are included.

### Data Availability

The data that support the findings of this study are available from the corresponding author upon request.
